# Knowledge levels regarding antibiotics and their use among horse owners in the State of Rio Grande do Norte, Brazil

**DOI:** 10.29374/2527-2179.bjvm000425

**Published:** 2025-05-30

**Authors:** Ingrid Raquel dos Santos Araújo, Emilson Lima de Brito, Ubiratan Pereira de Melo, Amanda Louíse Bittencourt Mariz, Mauricio Teixeira Cavalheiro, Cintia Ferreira, Leonardo Fiusa de Morais, Renato Fernandes de Souza

**Affiliations:** 1 Liga de Estudo em Medicina Interna Equina (Lamieq), Centro Universitário Maurício de Nassau, Natal, RN, Brazil.; 2 Centro Universitário Maurício de Nassau, Natal, RN, Brazil

**Keywords:** antimicrobial use, equine health, rational use of antimicrobials, veterinary prescriptions, uso de antimicrobianos, saúde equina, uso racional de antimicrobianos, prescrições veterinárias

## Abstract

This study aimed to evaluate the use of antimicrobials in horses, focusing on administration practices, adherence to veterinary prescriptions, and the impact on the development of antimicrobial resistance. The research was based on a questionnaire completed by 220 horse owners in the State of Rio Grande do Norte, Brazil. The data collected covered aspects including antibiotic use, prescription sources, the performance of culture and susceptibility tests, and the disposal of veterinary drug waste. The results indicated that 98.63% of horse owners administered antibiotics mainly to treat respiratory diseases. However, adherence to veterinary prescriptions was limited, with only 47.72% of horse owners correctly following the guidelines regarding dosage and treatment duration. Additionally, 68.18% of horse owners did not perform culture tests prior to antimicrobial treatment, and many obtained antibiotic recommendations from non-professional sources, such as friends or staff at veterinary product stores. These findings suggest that inappropriate antibiotic use practices, such as empirical and non-prescribed use, are contributing to the rise in antimicrobial resistance. The need for educational campaigns targeting horse owners is critical to raise awareness about the importance of rational antimicrobial use, risks of indiscriminate use, and necessity of performing laboratory tests to select appropriate treatment options.

## Resumo

Este estudo teve como objetivo avaliar o uso de antimicrobianos em equinos, com foco nas práticas de administração, adesão às prescrições veterinárias e o impacto no desenvolvimento de resistência antimicrobiana (AMR). A pesquisa foi conduzida por meio de uma análise de questionários aplicados a 220 proprietários de cavalos no estado do Rio Grande do Norte, Brasil. Os dados coletados abordaram aspectos como o uso de antibióticos, a origem das prescrições, a realização de exames de cultura e susceptibilidade, e o descarte de resíduos de medicamentos veterinários. Os resultados indicaram que 98,63% dos proprietários de cavalos utilizaram antibióticos, principalmente para doenças respiratórias, com uma adesão insuficiente às prescrições veterinárias: apenas 47,72% seguiram corretamente as orientações quanto à dosagem e duração do tratamento. Além disso, 68,18% dos entrevistados não realizaram exames de cultura antes do tratamento antimicrobiano, e muitos obtiveram recomendações de uso de antibióticos de fontes não profissionais, como amigos ou atendentes de lojas de produtos veterinários. Esses achados sugerem que práticas inadequadas de uso de antibióticos, como o uso empírico e sem prescrição, estão contribuindo para o aumento da resistência antimicrobiana. Destaca-se a necessidade de campanhas educativas direcionadas aos proprietários de equinos, visando conscientizá-los sobre a importância do uso racional de antimicrobianos, dos riscos do uso indiscriminado e da necessidade de realizar exames laboratoriais para seleção de tratamentos adequados.

## Introduction

Since the discovery of penicillin in 1928, antibacterial agents have revolutionized the treatment of diseases in veterinary and human medicine, leading to significant advancements in animal health. Over the years, these drugs have been continuously refined, resulting in increased specificity and efficacy against various pathogens (Silva et al., 2022; Tameirão et al., 2021).

However, the improper use of these medications in humans and animals has raised serious concerns within the scientific community due to the growing issue of bacterial resistance (Andrade et al., 2022; Tameirão et al., 2021). Antimicrobial resistance (AMR) resulting from inappropriate antibiotic use in animals also has implications for human health, as many classes of these drugs are shared across species, increasing the frequency of therapeutic failures that pose a significant risk to public and animal health (Martins et al., 2020; Sousa et al., 2019; Spricigo et al., 2024).

The emergence of multidrug-resistant bacterial strains is a global concern included in the “One Health” concept, representing a major threat to current strategies for the control and treatment of various infectious diseases in domestic animals. The indiscriminate use of antimicrobials in food-producing animals and athletic horses exacerbates bacterial resistance, creating a harmful cycle that limits available veterinary therapeutic options (Motta et al., 2017; Savariz et al., 2021; Silva et al., 2022; Spricigo et al., 2024).

The environmental impact of antibiotics used in veterinary medicine is significant. Improper disposal of these drugs into the environment contributes to the selection of resistant bacteria, exacerbating AMR. This scenario highlights the importance of a responsible and informed approach to antimicrobial use in veterinary medicine, emphasizing the prevention and proper management of infections, as well as the safe disposal of pharmaceutical waste, to safeguard animal, human, and environmental health (Silva et al., 2022).

AMR is a global issue, and understanding the molecular basis of resistance acquisition and transmission can contribute to the development of strategies to combat this issue. Additionally, a zoonotic dimension of AMR has been recognized. Horses can act as reservoirs for antibiotic-resistant organisms and resistance genes, which can affect veterinary treatments, impact animal welfare, and have economic implications. Such resistance can persist even in the absence of selective pressure. Furthermore, the use of antimicrobials in animals can promote resistance genes that pose future threats to human health by compromising the ability to treat infections. The common use of antimicrobials in equines, the close contact between humans and horses, and the associated risks and consequences to human health and therapy need to be re-evaluated (Ahmed et al., 2010; Melo et al., 2024; Tameirão et al., 2021).

Antibiotics continue to be a crucial tool in the treatment of infectious diseases in animals, with three distinct circumstances for their use, including treatment, metaphylaxis or control, and prophylaxis or prevention. In all cases where antibiotic administration is necessary, it should be prescribed following an appropriate diagnosis by a veterinarian, preferably with a thorough understanding of infectious disease epidemiology and pharmacological properties of the chosen antibiotic (Carvalho et al., 2017; Melo & Ferreira, 2022a; Melo et al., 2024; Simjee & Ippolito, 2022; Souza et al., 2024).

Although the prescription of antibiotics is the sole responsibility of veterinarians, various reports highlight the empirical use of these medications by horse owners without a proper clinical examination and disease diagnosis (Melo & Ferreira, 2021, 2022b; Melo et al., 2024). This practice, often occurring without professional supervision, can lead to several negative consequences, including the selection of resistant pathogens and potential exacerbation of the animal’s clinical conditions.

To the best of our knowledge, no specific studies have evaluated the level of knowledge regarding antibiotic use among horse owners in Brazil. Most available research focuses on bacterial isolation and AMR patterns in horses and other animals, without directly assessing the owners’ knowledge of the use of antimicrobials. The present study aimed to evaluate the practices and knowledge levels regarding antibiotic use among horse owners in the State of Rio Grande do Norte, Brazil. Analyzing these practices and owners’ understanding of appropriate antibiotic use will help identify knowledge gaps and inadequate practices, contributing to the development of educational strategies and interventions to improve antimicrobial management and combat bacterial resistance in the region.

## Materials and methods

A cross-sectional study was conducted including horse owners in the State of Rio Grande do Norte between September 2023 and June 2024. Those who agreed to participate were informed about the purpose of the research and were assured of the confidentiality of their identities, their animals’ identities, and other collected data. A structured questionnaire was administered, containing closed-ended questions regarding the owners’ education level, knowledge about antibiotics, previous use of antibiotics, source of antibiotic prescriptions, criteria for choosing antibiotics, duration of treatment prescribed by the veterinarian or initiated empirically, decisions regarding the discontinuation of empirical antibiotic therapy, disposal of empty and expired medication containers, and the primary diseases for which antibiotics were used. Additionally, knowledge about bacterial resistance was assessed through questions such as: “are antibiotics effective against bacteria?,” “are antibiotics effective against viruses?,” “can antibiotics have negative effects on the health of horses?,” “can bacteria become resistant to antibiotics in horses?,” “is bacterial resistance to antibiotics a problem in horses?,” “is bacterial resistance to antibiotics a problem in humans?,” “can bacterial resistance to antibiotics spread from humans to horses?,” and “can bacterial resistance to antibiotics spread from horses to humans?.”

Following the acquisition of oral and/or written consent, the questionnaires were administered individually using a convenient sampling framework. Owners completed the questionnaire independently, and assistance was provided only in case of illiteracy or physical disability. In such cases, one of the authors assisted in completing the questionnaire without influencing the responses, following the methodology described by Medeiros et al. (2023).

The data were compiled in Microsoft Excel® 2013 spreadsheets for subsequent calculation of absolute and relative frequencies. Statistical analysis was performed using IBM SPSS Statistics software. Pearson’s chi-squared test (χ2), with a significance level of 5%, was used to evaluate the association between the owners’ education level and responses obtained.

## Results

This study included 220 horse owners. Among them, 8.63% (19/220) had not completed basic education, 6.81% (15/220) had completed basic education, 15% (33/220) had not completed high school education, 26.81% (59/220) had completed high school education, 20.9% (46/220) had not completed higher education, and 21.81% (48/220) had completed higher education.

Regarding the level of knowledge about antibiotics (Table 1), 69.09% (152/220) of the owners reported being familiar with the term “antibiotic” and its use in horses, whereas 30.91% (68/220) stated they were unfamiliar with the term. Among those familiar with the term, the highest percentage was observed among those who completed higher education (31.1%; 47/152), whereas the lowest percentage was found among owners who had not completed basic education (5.9%; 9/152) (p<0.005).

**Table 1:** Knowledge of horse owners in the state of Rio Grande do Norte about antibiotics, information-seeking behavior, and prior use.

1) Knowledge about antibiotics	Incomplete Elementary Education	Complete Elementary Education	Incomplete Secondary Education	Complete Secondary Education	Incomplete Higher Education	Complete Higher Education	*p-value*
a) Are you familiar with the term “antibiotics” and their use in horses?							
*Yes (152/220)*	5.9% (9)	6.1% (10)	5.3% (8)	25.8% (39)	25.8% (39)	31.1% (47)	0.000
*No (68/220)*	14.9% (10)	6% (5)	37.3% (25)	26.9% (20)	10.4% (7)	1.5% (1)	
							
b) How is information about the use of antibiotics in horses obtained?							
*Veterinarian (133/220)*	5.3% (7)	3.8% (5)	13.5% (18)	31.6% (42)	22.6% (30)	23.3% (31)	0.000
*Internet (8/220)*	37.5% (3)	0% (0)	0% (0)	0% (0)	62.5% (5)	0% (0)	
*Friends/other owners (65/220)*	13.8% (9)	15.4 (10)	23.1% (15)	26.2 (17)	10.8% (7)	10.8% (7)	
*Books/formal education (14/220)*	0% (0)	0% (0)	0% (0)	0% (0)	28.6% (4)	71.4% (10)	
							
2) Previous use of antibiotics							
a) Have your horse(s) been treated with antibiotics before?							
*Yes (217/220)*	8.5% (19)	6.1% (15)	15.5% (33)	26.8% (58)	21.1% (46)	22.1% (48)	0.243
*No (3/220)*	0% (0)	0% (0)	0% (0)	33.3% (1)	33.3% (1)	33.3% (1)	
							
b) If yes, when was the last time one of your horses received antibiotics?							
*In the last 6 months (135/220)*	11.1% (15)	8.1% (11)	19.3% (26)	32.6% (44)	14.8% (20)	14.1% (19)	0.017
*6–12 months ago (35/220)*	11.45 (4)	2.9% (1)	11.4% (4)	17.1% (6)	28.6% (10)	28.6% (10)	
*13–24 months ago (37/220)*	0% (0)	5.4% (2)	2.7% (1)	18.9% (7)	32.4% (12)	40.5% (15)	
*More than 25 months ago (10/220)*	0% (0)	10% (1)	20% (2)	10% (1)	30% (3)	30% (3)	
*Never (3/220)*	0% (0)	0% (0)	0% (0)	33.3% (1)	33.3% (1)	33.3% (1)	

When asked about the source of information regarding the use of antibiotics, 60.45% (133/220) of the owners indicated that they obtained information from veterinarians, whereas 29.54% (65/220) reported obtaining information from friends or other horse owners. A total of 98.63% (217/220) of the interviewed owners stated that they had used antibiotics previously, but no difference was observed across the different educational levels (p>0.005). Among the owners who responded positively, 61.36% (135/220) reported using antibiotics within the last 6 months.

**Figure 1:**
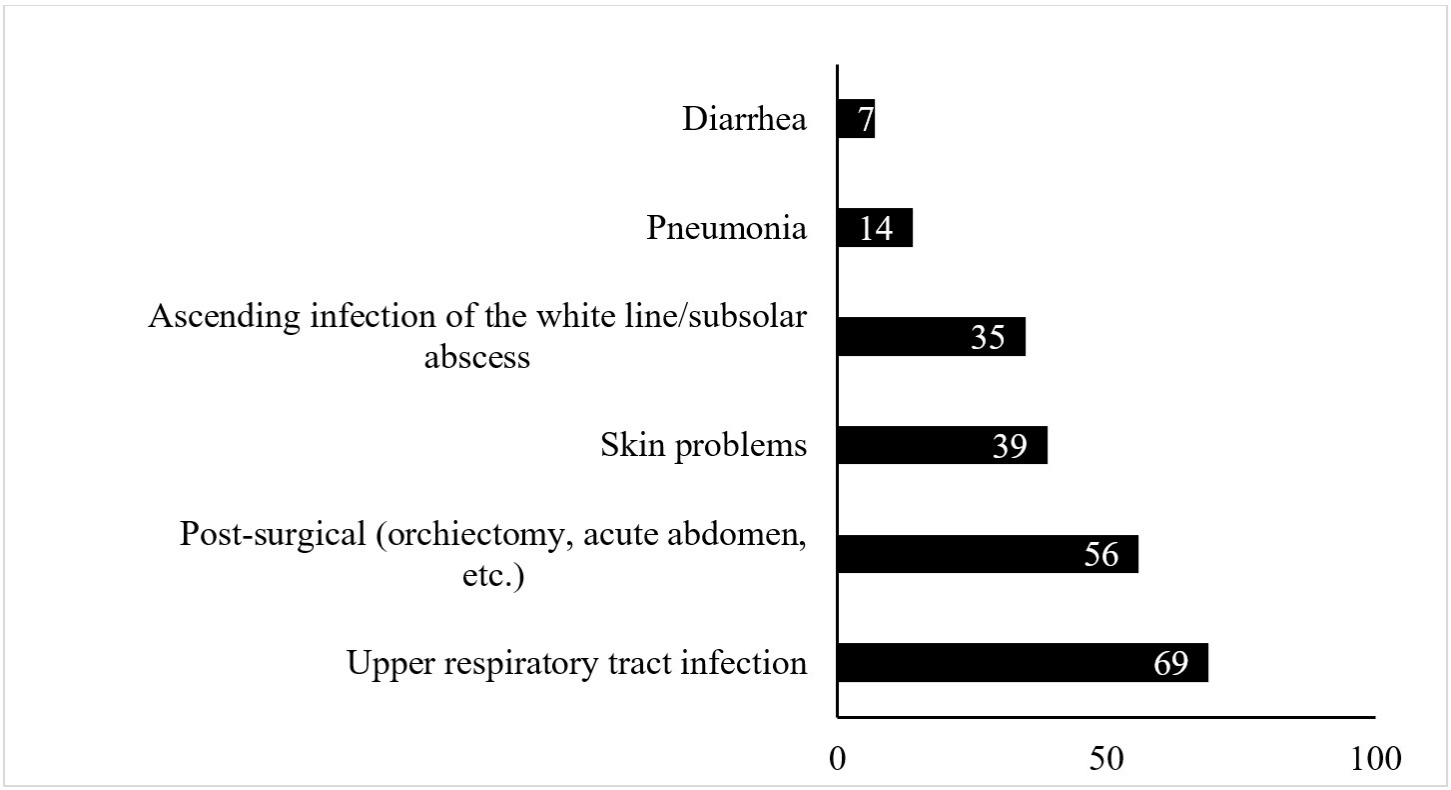
Main diseases in horses for which owners reported the use of antibiotics.

Figure 1 illustrates the main conditions for which owners reported using antibiotic therapy. Among these, the most commonly cited reasons were upper respiratory tract infections (31.36%; 69/220) and post-surgical procedures such as orchiectomy and gastrointestinal surgeries (25.45%; 56/220). Other reported reasons included skin infections and subsolar abscesses.

When asked about the source of antibiotic prescriptions (Table 2), 47.72% (105/220) stated that they used antibiotics following the veterinarian’s prescription, whereas 26.36% (58/220) reported using antibiotics empirically. In addition, 20.45% (45/220) owners relied on recommendations from the clerks at veterinary product stores. Among those who reported using antibiotics only after a veterinary prescription, a higher proportion was observed among owners with a higher level of education (p<0.05).

**Table 2:** Source of prescription, selection criteria, and administration of antibiotics among horse owners in the state of Rio Grande do Norte.

1) Source of antibiotic prescription	Incomplete Elementary Education	Complete Elementary Education	Incomplete Secondary Education	Complete Secondary Education	Incomplete Higher Education	Complete Higher Education	*p-value*
When your horse(s) receive antibiotics, the administration is based on:							
*Veterinary Prescription (105/220)*	3.8% (4)	3.8% (4)	9.5% (10)	23.8% (25)	23.8% (25)	35.3% (37)	0.000
*Recommendation from a friend (10/220)*	0% (0)	0% (0)	0% (0)	80% (8)	20% (2)	0% (0)	
*Store clerk at the veterinary products store (45/220)*	20% (9)	15.6% (7)	35.6% (16)	20% (9)	4.4% (2)	4.4% (2)	
*Empirical use (on your own, without veterinary prescription) (58/220)*	10.7% (6)	3.6% (2)	12.5% (7)	28.6% (17)	28.5% (17)	16.1% (9)	
*Empirical use (staff administer without my knowledge) (2/220)*	0% (0)	100% (2)	0% (0)	0% (0)	0% (0)	0% (0)	
							
2) Criteria for choosing antibiotics							
When antibiotic therapy is prescribed by a veterinarian, which criteria are generally considered, in your opinion?							
*Culture and sensitivity testing (11/220)*	11.1% (1)	0% (0)	0% (0)	33.3% (4)	0% (0)	55.6% (6)	
*Previous successful experience (41/220)*	10% (4)	7.5% (3)	15% (6)	25% (10)	25% (10)	17.5% (8)	
*Severity of the clinical condition (10/220)*	0% (0)	0% (0)	0% (0)	50% (5)	30% (3)	20% (2)	0.000
*Empirical knowledge about antibiotics (29/220)*	7.1% (2)	3.6% (1)	0% (0)	14.3% (5)	50% (14)	25% (7)	
*General knowledge of the veterinarian about antibiotics (129/220)*	9.4% (12)	8.7% (11)	21.3% (27)	26.8 (35)	15% (19)	18.8% (25)	
							
3) Administration of Antibiotics:							
Do you always follow the veterinarian’s instructions when administering antibiotics?							
*Yes (70/220)*	1.5% (1)	1.5% (1)	2.9% (2)	19.1% (14)	23.5% (17)	51.5% (35)	0.000
*No (150/220)*	12.2% (18)	9.5% (14)	20.9% (31)	29.7% (45)	18.9% (29)	8.8% (13)	

Regarding owner’s perception about the primary criteria used by attending veterinarians for selecting antibiotics were the knowledge level of the professional (58.63%; 129/220) and previous successful experiences with certain antibiotics (18.63%; 41/220). When asked if they followed the veterinarian’s recommendations regarding the correct use of antibiotics, 68.18% (150/220) admitted that they did not comply.

**Figure 2:**
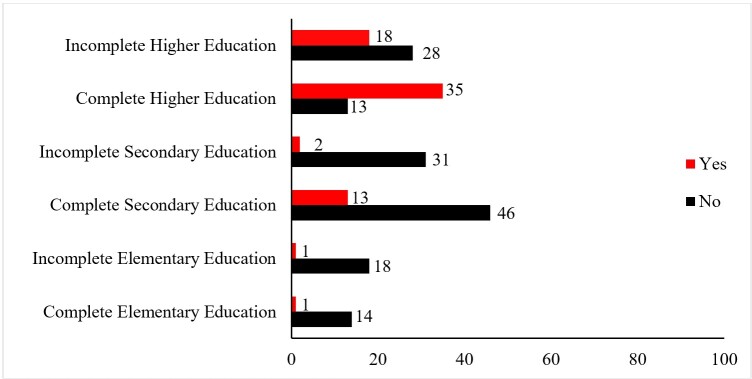
Veterinary practitioners’ reports on discussing antimicrobial susceptibility testing with horse owners.

The owners were asked whether the attending veterinarians had previously informed them about the use of culture and sensitivity testing to guide antibiotic selection (Fig. 2), and 68.18% (150/220) responded negatively. Regarding the duration of treatment prescribed by the attending veterinarians (Table 3), 40.90% (90/220) of the owners stated that it varied according to the diagnosis. However, when antibiotics were used empirically, 45% (99/220) of the owners reported limiting use up to 5 days.

**Table 3:** Treatment duration when antibiotics are prescribed by veterinarians or empirically used by horse owners in the state of Rio Grande do Norte.

1) Duration of treatment prescribed by the veterinarian	Incomplete Elementary Education	Complete Elementary Education	Incomplete Secondary Education	Complete Secondary Education	Incomplete Higher Education	Complete Higher Education	*p-value*
When antibiotic treatment is prescribed by a veterinarian, for how many days, on average, does the veterinarian typically recommend the treatment?							
*Less than 5 days (17/220)*	6.2% (1)	6.2% (1)	31.2% (5)	37.5% (7)	18.9% (3)	0% (0)	
*5 days (58/220)*	10.5% (6)	0% (0)	17.5% (10)	28.1% (17)	22.8% (13)	21.1% (12)	
*7 days (40/220)*	15% (6)	5% (2)	17.5% (7)	22.5% (9)	12.5% (5)	27.5% (11)	0.000
*More than 7 days (9/220)*	44.5% (4)	0% (0)	0% (0)	0% (0)	22.2% (2)	33.3% (3)	
*Varies with the disease (90/220)*	2.2% (2)	13.2% (12)	8.9% (8)	25.6% (23)	25.6% (23)	24.5% (22)	
*I do not have this information (6/220)*	0% (0)	0% (0)	50% (3)	50% (3)	0% (0)	0% (0)	
							
2) Duration of empirical treatment:							
When you administer antibiotics empirically (on your own), for how many days do you generally continue the treatment?							
*Less than 5 days (99/220)*	18.2% (18)	10.1% (10)	20.2% (20)	32.3% (32)	17.2% (17)	2% (2)	
*5 days (39/220)*	2.6% (1)	0% (0)	7.9% (3)	28.9% (12)	31.7% (12)	28.9% (11)	
*7 days (27/220)*	0% (0)	3.8% (1)	0% (0)	19.2% (5)	30.8% (8)	46.2% (13)	0.000
*More than 7 days (10/220)*	0% (0)	0% (0)	0% (0)	10% (1)	10% (1)	80% (8)	
*No defined pattern (45/220)*	0% (0)	9.1% (4)	22.7% (10)	20.5% (9)	18.2% (8)	29.5% (14)	
							
3) Decision to interrupt empirical antibiotic therapy:							
How do you decide when to stop the treatment?							
*Clear improvement in clinical signs (87/220)*	9.5% (8)	9.5% (8)	13.1% (11)	40.5% (35)	14.3% (12)	13.1% (13)	
*Pre-determined time (88/220)*	0% (0)	3.5% (3)	9.3% (8)	17.4% (15)	31.4% (27)	38.4% (35)	0.000
*Recommendation from other owners (44/220)*	25% (11)	9.1% (4)	31.8% (14)	20.5% (9)	13.6% (6)	0% (0)	
*Online recommendations (1/220)*	0% (0)	0% (0)	0% (0)	0% (0)	100% (1)	0% (0)	

**Figure 3:**
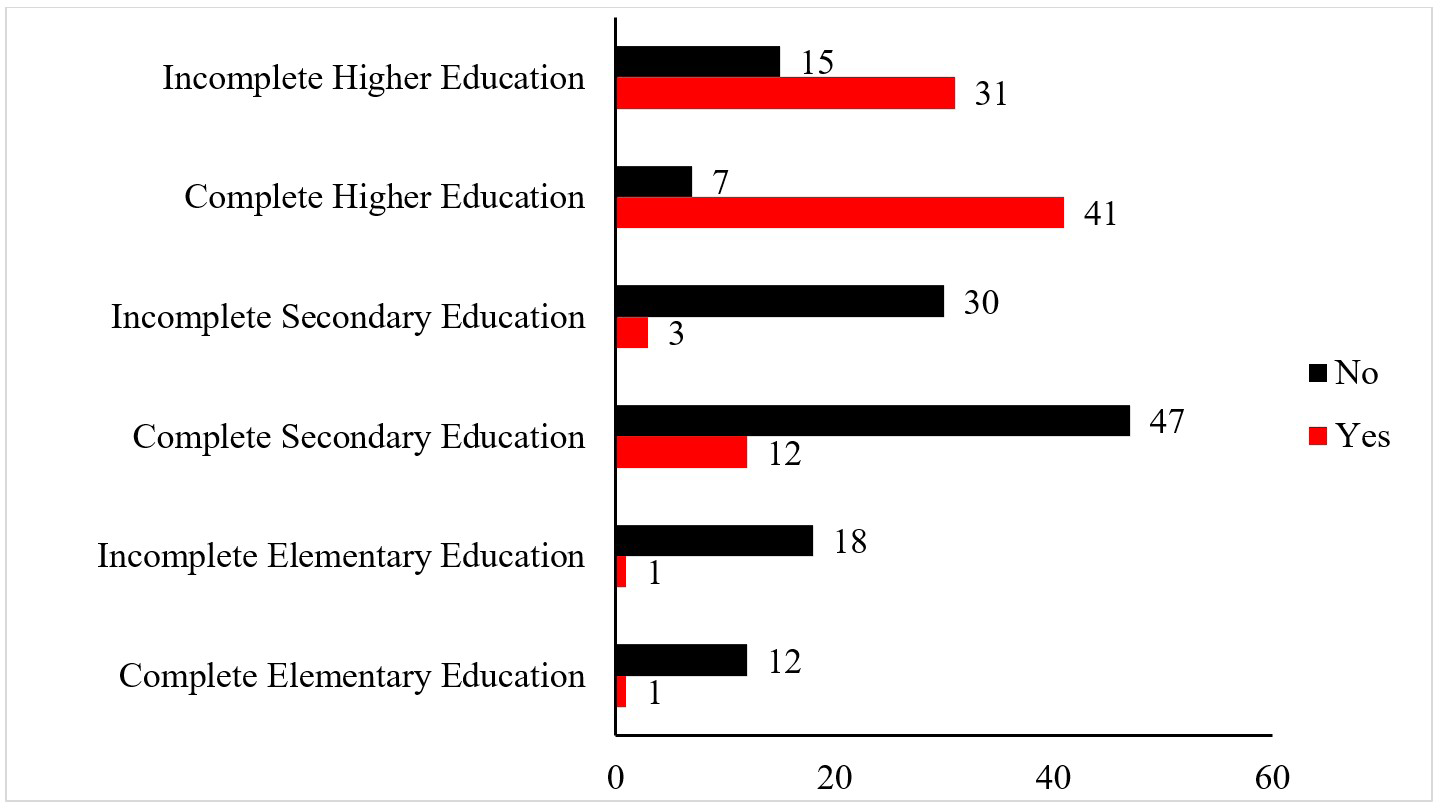
Awareness of antimicrobial resistance among horse owners in the state of Rio Grande do Norte, Brazil.

When asked about their knowledge of AMR (Fig. 3), 129 owners (58.63%) responded that they were unaware of its existence. The highest levels of AMR awareness were found among owners with completed or incomplete higher education.

When asked how the owners determined the appropriate antibiotic dosage (Table 4), 38.18% (84/220) responded that they relied on experience from previous treatments, whereas 32.27% (71/220) followed the veterinarian’s instructions.

**Table 4:** Determination of antibiotic dosage and disposal of empty antibiotic vials by horse owners in the state of Rio Grande do Norte.

1) Determination of antibiotic dosage	Incomplete Elementary Education	Complete Elementary Education	Incomplete Secondary Education	Complete Secondary Education	Incomplete Higher Education	Complete Higher Education	*p-value*
When administering antibiotics to your horses, how do you determine the dose to be given?							
*Strictly following the veterinarian’s instructions (71/220)*	2.9% (2)	5.7% (4)	8.6% (6)	25.7% (19)	15.7% (11)	41.4% (29)	0.000
*Based on the information on the medication label (58/220)*	5.3% (3)	3.5% (2)	3.5% (2)	19.3% (12)	40.4% (23)	28.1% (16)	
*Consulting other sources, such as the internet (2/220)*	0% (0)	0% (0)	50% (1)	50% (1)	0% (0)	0% (0)	
*Using experience from previous treatments (84/220)*	16.7% (14)	10.7% (9)	25% (21)	29.7% (25)	14.3% (12)	3.6% (3)	
*Financial costs (5/220)*	0% (0)	0% (0)	60% (3)	40% (2)	0% (0)	0% (0)	
							
2) Disposal of empty and expired medication bottles							
How do you typically dispose of empty and expired medication vials?							
*Selective waste collection (35/220)*	0% (0)	0% (0)	5.7% (2)	25.7% (9)	22.9% (8)	45.7% (16)	0.000
*Common trash (135/220)*	10.4% (14)	5.9% (8)	20.7% (28)	28.1% (38)	20.7% (28)	14.1% (19)	
*Return to the veterinarian/store (8/220)*	0% (0)	0% (0)	0% (0)	25% (2)	37.5% (3)	37.5% (3)	
*Incineration (11/220)*	0% (0)	0% (0)	0% (0)	27.3% (3)	18.2% (2)	54.5% (6)	
*Landfilling on the property (31/220)*	16.1% (5)	22.6% (7)	9.7% (3)	22.6% (7)	16.1% (5)	12.9% (4)	

Regarding the disposal of empty and expired medication bottles (Table 4), 61.36% (135/220) of owners reported discarding them with regular household waste, whereas 15.90% (35/220) reported using selective collection for hospital-origin products. Additionally, 14.09% of the owners disposed of the materials in landfills located on their own property.

**Figure 4:**
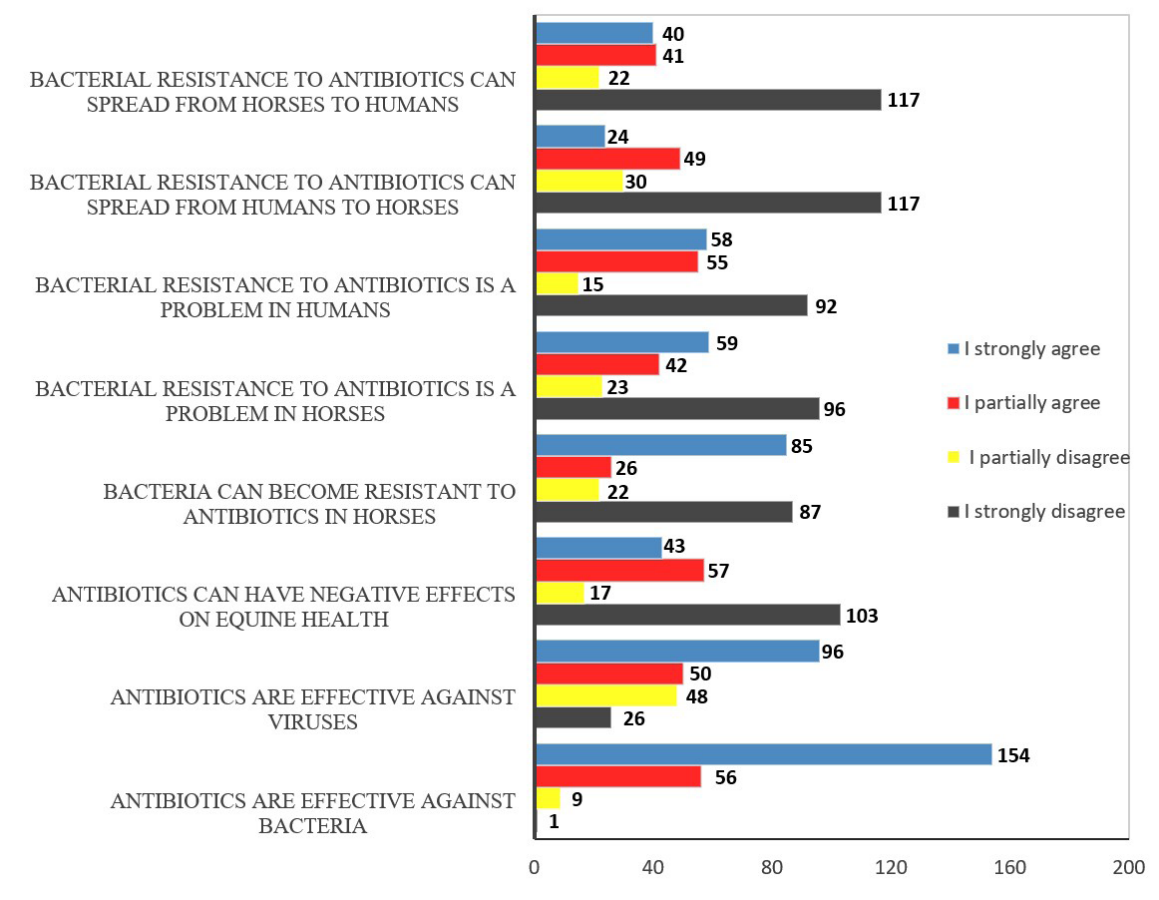
Level of Perception of Horse Owners in Rio Grande do Norte on the Effectiveness of Antibiotics and the Potential Development of Antimicrobial Resistance.

Regarding knowledge of antibiotic efficacy and resistance, 70% (154/220) of the owners correctly stated that antibiotics are effective against bacteria (Fig. 4). However, a majority of the interviewed owners (53.18%; 117/220) disagreed with the statement that AMR can spread from horses to humans or from humans to horses. Additionally, 43.63% (97/220) of the owners strongly disagreed with the statement that “antibiotic-resistant bacterial infections are a problem in horses.”

## Discussion

Horses are susceptible to a wide range of infectious diseases, most of which are caused by bacterial pathogens. While antibiotics are essential for treating existing bacterial infections, the effective prevention of such infections depends on other factors, such as biosecurity and prophylaxis using vaccines or antibiotics. The development of AMR is mainly due to the overuse and misuse of clinically essential antibiotics, as well as lack of evidence-based diagnoses and antibiotic treatment without a prescription (Kabir et al., 2024).

The increasing prevalence of AMR in equines presents serious challenges for animal and human health, as horses serve as reservoirs for zoonotic bacteria. Furthermore, the emergence of multi-drug-resistant (MDR) bacteria, which are resistant to three or more classes of antibiotics, exacerbates the condition and complicates treatment. Several studies in Brazil have reported MDR pathogens in equines (Braga et al., 2023; Melo & Ferreira, 2022b; Motta et al., 2017; Veiga et al., 2024).

Corroborating the findings of Taylor and Scallan Walter (2023), our study demonstrated that many horse owners lack a good understanding of AMR, and most are unaware of the potential impact of antibiotic use in horses on human health. While educating owners on the judicious antibiotic use is necessary, less than half of the interviewed owners reported adherence to veterinary prescriptions, which is in contrast to the findings from a study involving pet owners (Taylor & Scallan Walter, 2023). These findings suggest that veterinarians can promote and reinforce behaviors aligned with appropriate antibiotic use through client education and active involvement in the prescribing process.

In the present study, 98.63% of horse owners indicated that they have used antibiotics, primarily for treating respiratory diseases. This high percentage may reflect both overuse and misuse, as only 47.72% of the interviewed owners administered antibiotics based on veterinary prescriptions. The practice of using antibiotics without a veterinary prescription, following a thorough clinical examination, jeopardizes equine health and increases the likelihood of developing AMR. This practice may contribute to the selection of resistant bacterial strains, ultimately exacerbating the global challenge of AMR in animal and human populations.

Decisions regarding antimicrobial use in veterinary medicine are shaped by a complex interplay of medical, social, and behavioral factors that involves various stakeholders, including animal owners. Animal owners are responsible for monitoring their animals’ health, seeking and financing medical care when necessary, and administering treatments as prescribed by veterinarians (Taylor & Scallan Walter, 2023). However, our results demonstrated that 68.18% of owners, when obtaining antibiotic prescriptions after a clinical examination, did not follow veterinarians’ recommendations regarding dosage and treatment duration. Furthermore, obtaining “treatment protocols” based on recommendations from a friend or veterinary product store clerk, along with empirical use, are common practices that may significantly contribute to AMR and reflect misuse of these drugs in the studied population.

Appropriately collected samples from infection sites should be cultured and susceptibility testing of bacterial isolates should be performed, whenever possible, before initiating antimicrobial treatment. These practices are required for the use of antimicrobials in many countries (Wilson & Magdesian, 2021). However, our findings showed that 68.18% of owners reported that attending veterinarians did not discuss the performance of cultures for selecting the most appropriate antimicrobials. By assisting in the selection of the appropriate antimicrobial and dosing regimen, culture and susceptibility testing help maximize the therapeutic success at the individual equine level and reduce the acquisition of resistance among bacterial isolates affecting equines at the population level (Wilson & Magdesian, 2021).

Clinicians should become familiar with antimicrobial susceptibility patterns of isolates in their practice area, and work with their microbiology laboratories to update information every few years. Antibacterial susceptibility profiles of bacteria, such as β-hemolytic *Streptococcus* spp., *Actinobacillus* spp., and anaerobes, excluding *Bacteroides* spp., are somewhat predictable and show limited geographical variation. In contrast, *Enterobacteriales*, *Pseudomonas* spp., *Bordetella* spp., *Enterococcus* spp., coagulase-positive *Staphylococcus* spp., α-hemolytic *Streptococcus* spp., *B. fragilis*, and, more recently, *R. equi*, have either unpredictable susceptibility or are predictably resistant to particular antibiotics or classes of antibiotics. Thus, performing susceptibility tests on these isolates is particularly important (Wilson & Magdesian, 2021). These guidelines are useful in areas or countries where microbial culture and susceptibility testing are routinely performed in clinical practice; however, to our knowledge, such practices are not common facts supported by the high percentage of owners who reported being uninformed about the performance of these tests.

The judicious use of antimicrobials is broadly defined as selecting the optimal type, dose, and duration of antimicrobial treatment while reducing inappropriate and excessive use, with the goal of achieving the best possible clinical outcome and minimizing the emergence of AMR (Redding & Cole, 2019). In this context, our study demonstrated a wide variation in the duration of antimicrobial administration, especially when empirically administered by the owner (Table 3), which may have contributed to the misuse of these drugs.

Inappropriate administration of antimicrobials by owners can occur in various ways, such as early cessation of treatment due to perceived recovery, failure to follow administration instructions, non-compliance with prescribed schedules, abandonment of treatment when animals resist administration, use of leftover drugs from previous prescriptions when animals experience recurrences of a chronic condition, or the use of human medications in animals (Redding & Cole, 2019). Among these factors, the early cessation of treatment due to perceived recovery was identified as a key determinant for treatment interruption.

To reduce unnecessary use of antimicrobials, veterinarians must balance the abstract phenomenon of AMR with the immediate concerns related to the animal’s health and well-being, as well as the owners’ concerns for their pets. Prescribing antimicrobials solely to satisfy owners or minimize risks (i.e., prescribing “just in case”) is considered a key barrier to achieving effective antimicrobial stewardship (Redding & Cole, 2019).

In the present study, 61.36% (135/220) of owners reported disposing of expired or empty antimicrobial containers in regular household waste. This rate exceeds that reported by Gottardo et al. (2021), who interviewed 22 rural producers in the municipality of Sete de Setembro, located in the Northwest region of Rio Grande do Sul. In their study, the primary disposal methods for expired and empty antimicrobial containers were disposal in regular household waste (50%) and burial on rural property (40%). Additionally, most producers in Gottardo et al. (2021) study stated that they had never received information on the proper disposal of empty containers, expired medications, syringes, or needles.

These findings highlight the need for educational programs for equine owners to raise awareness about proper disposal practices for veterinary drug waste. The lack of adequate guidance may lead to improper disposal, as observed in this study, resulting in potential environmental and public health risks. Therefore, health authorities and veterinary organizations must develop and implement clear and accessible guidelines for the safe management of such waste.

Due to the intense movement of equines between various equestrian events held within the region or in distant states, the improper disposal of vials containing antibiotic residues used during these journeys may have a significantly broader impact (Silva et al., 2024).

The owners exhibited knowledge gaps regarding the effectiveness of antibiotics against viruses, the transmission of antibiotic-resistant organisms between humans and equines, and the potential side effects of antibiotics (Fig. 4). These findings align with those observed by Frey et al. (2024), who identified similar gaps among cat and dog owners when assessing their knowledge and preferences for veterinary communication. These knowledge gaps present opportunities for veterinarians and their teams to provide training and information to their clients (Frey et al., 2024).

Given the lack of awareness observed among some horse owners about the side effects of antimicrobial medications, it is crucial that veterinarians clearly communicate the risks and benefits of antibiotic use (Frey et al., 2024). This includes discussing potential side effects such as intestinal dysbiosis, hemolytic anemia, and hypersensitivity reactions.

Despite the findings highlighting the need for greater awareness regarding the rational use of antibiotics, this study has some limitations. The cross-sectional design captures only the perceptions of horse owners at a specific time point. Additionally, the interviewed owners may have interpreted certain survey questions regarding “antimicrobial resistance” or “veterinary prescription” differently, which may have impacted their responses. Moreover, whether the survey sample is representative of all horse owners in the State of Rio Grande do Norte is unclear; therefore, the results may not be generalizable to horse owners from other Brazilian states or nationwide. Thus, a more comprehensive study involving horse owners from different states is needed.

Another factor that may have led to a bias in the results is the financial condition of the horse owners, as treatment costs can impact the correct use of antibiotics. A social desirability bias may also have influenced the results if owners selected responses they believed to be more acceptable rather than selecting responses that represented their actual knowledge and attitudes.

Recognizing the descriptive nature of this assessment and the inherent limitations of cross-sectional studies and convenience sampling, further research is needed to understand the factors that drive owners’ perceptions of antibiotic use and AMR and identify the difference in the level of knowledge and attitudes among owners of different equine modalities.

The cross-sectional design of this study represents a notable limitation, as it captures only a snapshot of the attitudes and behaviors of the horse owners without allowing for the analysis of trends over time. This approach does not allow for the inference of causality, which is crucial for understanding how antimicrobial use may influence the development of AMR in the long term. Therefore, the use of a longitudinal study design could provide a more dynamic perspective, allowing for the evaluation of changes in owners’ practices and attitudes and facilitating the identification of effective interventions.

Although this study provides valuable preliminary data on the use of antimicrobials among horse owners, its methodological limitations necessitate cautious interpretation of the findings. To strengthen the validity of the study findings and broaden the applicability of the data, future research should incorporate more robust methodological approaches, such as stratified sampling, the use of microbiological data, and longitudinal analysis. These improvements would yield a deeper understanding of the relationship between antimicrobial use and AMR in equines, significantly advancing the knowledge in the field of veterinary public health.

The absence of clinical and microbiological data represents a significant limitation of this study, especially in the context of the State of Rio Grande do Norte, where many horse owners use antimicrobials empirically, without proper veterinary guidance or laboratory testing. Moreover, the topic of microbiological testing is rarely addressed in veterinarian–horse owner discussion in the region, which further complicates obtaining objective information on antimicrobial use practices. To enhance the credibility and robustness of the results, future studies should incorporate microbiological analyses, allowing for a more accurate and objective assessment of the impact of empirical antimicrobial use on the observed resistance.

## Conclusion

The analysis of antimicrobial use in horses revealed inadequate practices that contributed to the increase in AMR, such as empirical use and lack of adherence to veterinary prescriptions. The high rate of antibiotic use, mainly for respiratory diseases, coupled with the limited use of culture and susceptibility testing highlights the urgent need for educational strategies for horse owners. Public awareness campaigns should emphasize the importance of rational use of antimicrobials, strict adherence to veterinary prescriptions, and conducting laboratory testing for appropriate treatment selection. Only through awareness and changes in management practices can we mitigate the impact of AMR and ensure the effectiveness of antibiotic therapies in the future.
